# Microhemorrhages and Siderosis in Cognitive Impairment

**DOI:** 10.1002/alz.089750

**Published:** 2025-01-09

**Authors:** Hessah A Alotibi

**Affiliations:** ^1^ University Health Network Toronto, Toronto, ON Canada

## Abstract

**Background:**

Microhemorrhages and superficial siderosis (SS) have been reported in patients with mild cognitive impairment (MCI) and dementia, especially in the context of Alzheimer’s disease (AD) and cerebrovascular disease. Cerebral amyloid angiopathy and small vessel disease (SVD) have both been implicated in microhemorrhages and SS but their prevalence in those with MCI and dementia and their relationship to SVD is unknown.

**Method:**

We conducted a retrospective chart review of patients with MCI or dementia that had undergone MRI scans from 2014 To 2023. We calculated prevalence of microhemorrhages and SS, analyzed the distribution patterns, and assessed small vessel ischemic changes using the Fazekas scale. Exclusions included traumatic brain injury, large stroke, and subjective cognitive impairment.

**Result:**

2852 individuals underwent underwent an MRI for CI and 120 (4.2%, 95% CI: 3.5‐5%) patients had microhemorrhages and/or SS and fulfilled inclusion criteria (mean age 80.52 (9.1) years; 55.8% male). Of the patients with hemorrhagic findings, 73% had microhemorrhages (mean 7.51 microhemorrhages patient), 7.5% had SS and 19.2% exhibited both. The microhemorrhages were deep (25.8%), lobar (35.0%), and mixed (39.2%). Fazekas rating scale was 0 in 0.8% of patients with microhemorrhages, 1 in 34.2%, 2 in 44.2%, and 3 in 20.8%. The most prevalent diagnoses in patients with hemorrhagic findings were mixed vascular and AD dementia (34.2%), vascular CI (25.0 %), AD (18.3%), vascular MCI(7.5%), MCI due to possible AD (4.2%), Parkinson/Lewy Body dementia (5.0%), dementia NYD (4.2%), frontotemporal dementia (0.8%), and progressive supranuclear palsy (0.8%). Common comorbidities included hypertension (70.0%), dyslipidemia (50.0%), and ischemic heart disease (25.8%).

**Conclusion:**

4% of patients investigated for cognitive impairment have microhemorrhages and/or SS. Most of the patients had CI due to AD and/or vascular disease. Mild or moderate SVD as indicated by Fazekas scale was common as was hypertension. The etiology of microhemorrhages and SS is likely multi‐factorial and SVD may contribute to their presence, but hypertension may be an independent risk factor that warrants further investigation.

